# An Efficient Experimental Methodology for the Assessment of the Dynamic Behaviour of Resilient Elements

**DOI:** 10.3390/ma13132889

**Published:** 2020-06-27

**Authors:** Salvatore Reina, Robert Arcos, Arnau Clot, Jordi Romeu

**Affiliations:** 1 Acoustical and Mechanical Engineering Laboratory (LEAM), Universitat Politècnica de Catalunya (UPC), C/Colom 11, 08222 Terrassa, Barcelona, Spain; robert.arcos@upc.edu (R.A.); arnau.clot@upc.edu (A.C.); jordi.romeu@upc.edu (J.R.); 2 Department of Mechanical Engineering, Escuela Politécnica Nacional, Ladrón de Guevara E11-253, 17-01-2759 Quito, Ecuador; 3 Serra Húnter Fellow, Universitat Politècnica de Catalunya (UPC), 08222 Terrassa, Spain

**Keywords:** frequency response functions, sweep sine excitation, resilient elements, ISO 10846-3

## Abstract

The assessment of the dynamic behaviour of resilient elements can be performed using the indirect method as described in the standard ISO 10846-3. This paper presents a methodology for control the error on the estimation of the frequency response functions (FRF) required for the application of the indirect method when sweep sine excitation is used. Based on a simulation process, this methodology allows for the design of the sweep sine excitation parameters, i.e., the sweep rate and the force amplitude, to control three types of errors associated to the experimentally obtained FRF in the presence of background noise: a general error of the FRF in a selected frequency range, and the errors associated to the amplitude and the frequency of the FRF resonance peak. The signal processing method used can be also tested with this methodology. The methodology has been tested in the characterisation of two different resilient elements: an elastomer and a coil spring. The simulated error estimations has been found to be in good agreement with the errors found in the measured FRF. Furthermore, it is found that for large signal-to-noise ratios, both sweep rate and force amplitude significantly affect the FRF estimation error, while, for small signal-to-noise ratios, only the force amplitude can control the error efficiently. The current methodology is specially interesting for laboratory test rigs highly used for the dynamic characterisation of resilient elements which are required to operate efficiently, since it can be used for minimising test times and providing quality assurance. Moreover, the application of this methodology would be specially relevant when characterisation is done in noisy environments.

## 1. Introduction

Elastomeric materials are widely used as vibration control components in several types of structures. Some interesting applications can be found, for example, in railway tracks, where components such as rail pads or ballast mats are often implemented to mitigate railway-induced ground-borne vibrations [1,2,3]. Prior to its implementation, the isolation efficiency of a potential solution is often assessed using prediction methods. In these methods, the mechanical behaviour of the elastomeric materials is often represented using simplified viscoelastic models. Examples of these are the Kelvin–Voigt model, where the elastomeric element is represented using an elastic spring and a viscous damper [4], or the Maxwell model, where these components are connected in series [5]. Several alternative models have been considered in literature. Two examples of this are the use of state-dependent viscoelastic models to represent the dynamic behaviour of rail pads [6], or the use of a fitted generalised Kelvin–Voigt model in a recently proposed load-controlled testing approach, suitable for testing a large variety of viscoelastic materials [7]. Regardless of the type of model assumed, its associated mechanical parameters (i.e., stiffness and/or damping values) usually have to be determined from experimental measurements. In particular, these properties can be determined from either standard laboratory tests [8] or from in-situ experiments [9,10,11,12]. These methods, however, usually require some knowledge of the dynamic properties of the elastomeric material considered. These properties can be determined from either standard laboratory tests [8] or from in-situ experiments [9,10,11,12].

Both laboratory test rigs and in-situ experimental methods used for characterising the dynamic properties of elastomeric materials are commonly based on the experimental determination of the frequency response functions (FRF) of the system that includes the elastomeric material. Controlled dynamic excitation is usually used on this regard. Examples of this are the application of an impact excitation using instrumented hammers [13,14,15,16] or the excitation of the elastomeric material using a shaker device [17]. Background vibrations have been also considered for characterising the transmissibility (FRF that relates two vibration motions) of isolators [18].

Regarding laboratory testing methods, the international standard ISO 10846 describes three measurement methods for obtaining the dynamic stiffness of a linear elastic element: A driving point method [8], a direct method [19] and an indirect one [20]. Several authors have discussed the practical experimental difficulties found when applying ISO 10846. Morison et al. [21] suggested a method for correcting the contribution of the inertial forces of the tested system when using the driving point method. Ozgen et al. [22] proposed guidelines to avoid structural design issues of test systems, such as the effect of the used vibration fixtures or the Poisson’s effect on the material.

In the framework of the indirect method for the evaluation of the dynamic properties of a resilient material described in [20], shakers are commonly used as the excitation devices for the method implementation. Several types of excitations can be considered when using a shaker device. The most common examples are harmonic sines, linear or logarithmic sweep sines and random excitations. Regardless of the type of excitation considered, a good signal-to-noise ratio (SNR) is required to obtain reasonable estimates of the FRFs of a system [23] Therefore, an appropriate selection of the type of excitation used needs to consider both the spectral energy content of the excitation and the duration of exposure. In some applications, the sweep sine excitation is a good trade-off between both [24,25], although an incorrect selection of their parameters could lead to incorrect FRF estimations. Thus, the effect that the sweep rate of a sweep sine excitation has in the system response has been studied and discussed by several authors [24,26,27,28]. As system resonances are passed at a certain sweep velocity, their estimated amplitudes and frequencies differ from those expected to be obtained using harmonic excitation. Therefore, methods that assume a steady-state response for each excitation frequency, as it is the case of co-quad analysers, tracking filters or the use of the Hilbert transform, lead to wrong estimations of the system FRFs [24]. These distortions do not occur if the system FRFs are determined by processing methods based on Fourier transform to the entire measured time histories [24,28]. However, the accuracy of the estimated FRFs still depends on the method used for performing the time-frequency transform [27]. For example, the resonance amplitudes may be significantly underestimated if data reduction techniques are used [24]. Additionally, if the experiment cannot be carried out in a controlled environment (as it would be the case of in-situ approaches), the existing background noise may severely limit the accuracy of the estimated FRF.

This paper presents a methodology to define which excitation parameters should be used when measuring FRFs of a system using a sweep sine excitation in the context of the evaluation of dynamic stiffness of elastomeric materials or resilient elements. The methodology proposes to use a simplified analytical model of the testing system in order to quantify the errors associated with the selection of a particular sweep amplitude and sweep rate. Optimal values for the excitation parameters can be then obtained by finding the minimum amplitude and sweep rates that ensure a required accuracy. In particular, the methodology is applied to the case where the FRF of interest is the transmissibility required in the application of the ISO 10846-3 [20]. The paper is organised as follows: The proposed methodology for defining optimal experimental parameters for the characterisation of a resilient element is defined in Section 2. The experimental setup for the methodology validation is described in Section 3. Then, based on the described experimental setups, the methodology is applied to two case studies in Section 4.1 and Section 4.2. Finally, the discussion of the results obtained and the conclusions of this work are summarised in Section 5.

## 2. Methodology for Assessing the Dynamic Behaviour of Resilient Elements

**Figure 1:**
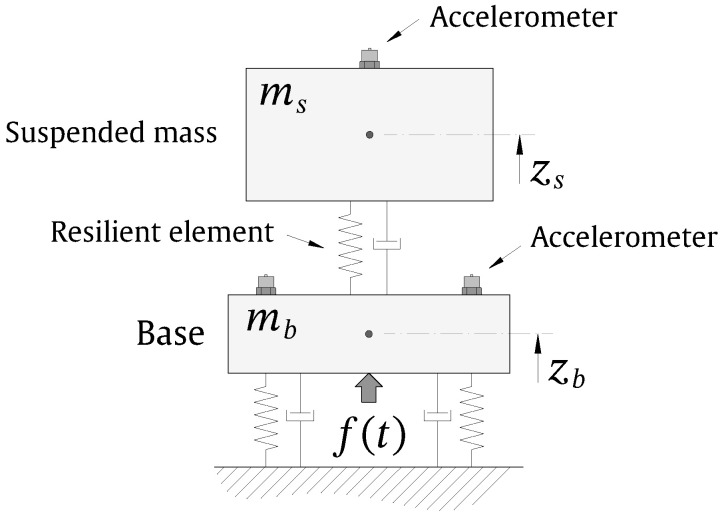
2DOF model of the laboratory test setup adopted. The big arrow represents the dynamic force applied by the shaker.

The indirect method for the evaluation of the axial dynamic properties of elastomeric materials or, in general, resilient elements proposed in the ISO 10846-3 [20] can be performed by various laboratory setups. In this work, a setup based on a two-degrees-of-freedom (2DOF) system, as presented in Figure 1, is adopted. This system consists of two masses: the base mass $m_{b}$, which rests on the ground through isolation springs of global stiffness and viscous damping $k_{b}$ and $c_{b}$, respectively; and the suspended mass $m_{s}$, which is resting on the resilient element to be studied. The dynamic stiffness and viscous damping of the resilient element are represented by $k_{e}$ and $c_{e}$ in this work. The system should be designed ensuring that both masses are vibrating solely in the vertical direction. The suspended mass has also the role of applying the required static preload to the resilient element. In the context of such a laboratory setup, the determination of the dynamic properties of the resilient element is obtained from the transmissibility between the vertical motion of these two masses due an external excitation f(t) vertically applied on $m_{b}$. In this work, the vertical motion of both masses is measured using a setup of accelerometers and the excitation is applied by a shaker. The external excitation applied by the shaker should be designed to ensure a linear behaviour of the resilient element [8] and to provide an accurate estimate of the transmissibility. The present work is based on the consideration that the excitation applied by the shaker is a linear sweep sine.

This article presents a methodology to design the parameters of the sweep sine excitation to control the error associated to the transmissibility estimation. This methodology is based on a simulation procedure to predict the error on the experimental estimation of the transmissibility in terms of the sweep sine parameters, the static stiffness of the resilient element, the mechanical characteristics of the test rig and the background noise.

### 2.1. Simulation of the Transmissibility in the Context of Sweep Sine Excitation

In order to simulate the response of a system such as the one appearing in Figure 1 due to the action of a linear sweep sine excitation, let us consider a sweep sine force applied by the shaker sweeping from an angular frequency of $\omega_{s}$ to $\omega_{e}$ defined by

(1) $$f\left(t\right)=F\sin\left(\frac{\alpha }{2}t^{2}+\omega_{s}t\right)$$

where *F* is the amplitude of the dynamic force applied by the shaker, α is the sweep rate and *t* is the time variable. The sweep rate in a linear sweep sine is defined by $\alpha =(\omega_{e}-\omega_{s})/T$. On the other hand, the response of the 2DOF system due to the action of an arbitrarily load is given by

(2) $$\begin{array}{cc} m_{b}{\ddot{z}}_{b} & =k_{e}\left(z_{s}-z_{b}\right)+c_{e}\left({\dot{z}}_{s}-{\dot{z}}_{b}\right)-k_{b}z_{b}-c_{b}{\dot{z}}_{b}+f\left(t\right)\\ m_{s}{\ddot{z}}_{s} & +k_{e}\left(z_{s}-z_{b}\right)+c_{e}\left({\dot{z}}_{s}-{\dot{z}}_{b}\right)=0 \end{array}$$

The response of the system governed by Equation (2) subjected to the action of the load in Equation (1) is proposed to be numerically computed by a time domain solver. Prior to that, the mechanical parameters of all the system should be known or estimated. Masses $m_{s}$ and $m_{b}$, as well as the stiffness and viscous damping $k_{b}$ and $c_{b}$ can be previously evaluated. To estimate the dynamic stiffness of the resilient element, it is proposed to use the static stiffness of the element $k_{e}^{s}$, which is usually previously evaluated in static loading laboratory tests. More precisely, the dynamic stiffness value is assumed to range between $k_{e}^{s}$ and $2k_{e}^{s}$ [29]. Regarding the damping of the resilient element, it is proposed to provide a lower limit of its estimation, since the errors on the experimental evaluation of the transmissibility are expected to be lower for higher values of the damping.

Once the time response of the system due the sweep sine excitation is simulated, in-situ background noise can be added to the signals to simulate a real laboratory scenario. Therefore, the simulated vibration response of the base ${\tilde{z}}_{b}={\tilde{z}}_{b}\left(t\right)$ can be written as [30]

(3) $${\tilde{z}}_{b}=z_{b}+\eta \left(t\right)$$

where the η(t) represents an experimental measurement of the background noise in the base of the test rig in normal (or preferable adverse) laboratory conditions.

The resulting time responses of the system due the sweep sine excitation, $z_{e}$ and ${\tilde{z}}_{b}$ are proposed to be processed with the same signal processing procedure that is intended to be used in the experimental evaluation of the transmissibility. Thus, this methodology can be also used to assess the signal processing method used. In this work, it is proposed to use the Welch’s method [31] with a 50% overlap, Hamming windows and considering eight overlapping segments.

### 2.2. Prediction of the Error Associated to the Transmissibility Experimental Estimation

To predict the error of the transmissibility experimental estimation, a parametric study is performed in which the theoretical transmissibility of the system is compared with the simulated one, computed following the steps described in the previous section. The theoretical transmissibility of the system $T_{sb}$ can be expressed as

(4) $$T_{sb}=\frac{Z_{s}}{Z_{b}}=\frac{k_{e}+i\omega c_{e}}{k_{e}+i\omega c_{e}-\omega^{2}m_{e}}$$

where $Z_{s}$ and $Z_{b}$ are the responses of both masses in the frequency domain, respectively. The parametric study accounts for three parameters: the dynamic stiffness of the elastomeric material (based on the range previously defined), the sweep rate α and the amplitude *F* of the sweep sine force. Sets of discrete values of each of these parameters within appropriate ranges should be defined. For each combination of parameters’ values, three types of errors are computed: A general error of the FRF ε, an error of the resonance frequency evaluation $\varepsilon_{f}$ and an error associated to the resonance amplitude evaluation $\varepsilon_{z}$.

The general error ε is defined in this methodology as

(5) $$\varepsilon =\frac{1}{N}\sum_{i=1}^{N}\left|\frac{T_{sb_{i}}-{\hat{T}}_{sb_{i}}}{T_{sb}^{\max}}\right|$$

where the hat notation is used to refer to simulated transmissibility, *i* is the index that follows sampling in frequency within a desired frequency range and *N* is the number of values in this range. The frequency range where this error is assessed is recommended to be selected containing the resonance of the transmissibility, since this is where the accuracy of the FRF is of most importance regarding dynamic stiffness assessment. The amplitude of the transmissibility at the resonance is represented by $T_{sb}^{\max}$ and the associated frequency as $f_{\max}$.

On the other hand, the error associated to the frequency where the resonance of the transmissibility is ocurring is defined by

(6) $$\varepsilon_{f}=\left|\frac{f_{\max}-{\hat{f}}_{\max}}{f_{\max}}\right|$$

while the error for the amplitude at the resonance is described by

(7) $$\varepsilon_{z}=\left|\frac{T_{sb}^{\max}-{\hat{T}}_{sb}^{\max}}{T_{sb}^{\max}}\right|$$

The methodology presented in this work proposes to design both the sweep rate and the dynamic load amplitude of the experiment using the numerical error predictions previously defined. Regarding the dynamic load, the range of valid amplitudes is defined by two limits: the lower one is defined by the minimum force that, when considered in the numerical calculations, predicts error values below the maximum acceptable error defined by the testing engineer; the upper one is defined by the maximum value in the range of force amplitudes that ensures a linear response of the resilient material. The best excitation amplitude will be the lower limit. Regarding the sweep rate, the best value will be the fastest rate that, when used in the numerical calculations, predicts error values below the maximum error accepted.

## 3. Experimental Setup for Methodology Validation

**Figure 2:**
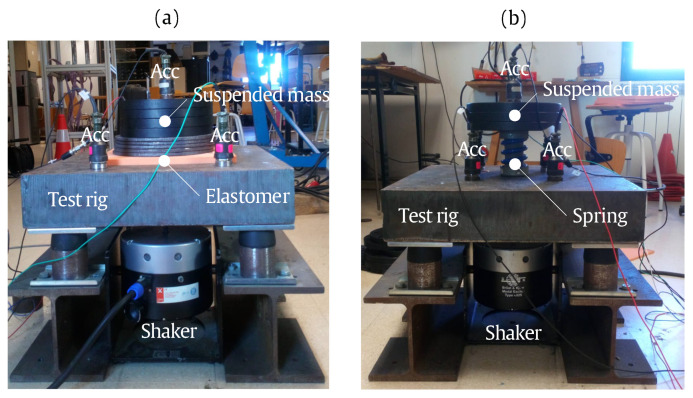
Experimental setup for the elastomeric element (**a**) and for the coil spring element (**b**).

In this section, the experimental setup considered for the validation of the proposed methodology is described. This experimental setup is constructed following the ISO 10846-3 standard [20] and is used in this paper to characterise two examples of resilient elements: an elastomer and a coil spring. In Figure 2, the experimental setups for both tested resilient elements are shown. Each experimental setup consists of a test rig, the tested resilient element, a suspended mass, a setup of accelerometers and the excitation shaker, the latter two linked to a signal acquisition hardware. The same test rig was used in both cases, consisting of by a steel block (as a base mass) supported through rubber vibration isolators by two parallel steel beams resting on the ground. The mechanical parameters of the test rig are defined in Table 1, together with the suspended masses used in each experimental setup. The static stiffnesses of both specimens were previously known from prior static tests and are also detailed in Table 1. The viscous damping coefficient defined as $\xi_{e}=c_{e}/\left(2\sqrt{k_{e}m_{s}}\right)$ is selected as a lower limit estimation of the real damping, following the methodology proposal. The excitation shaker used was a Brüel&Kjaer Type 4825 (Copenhagen, Denmark). The accelerometers setup was composed by five piezoelectric accelerometers (PCB Piezotronics, Type 393B31, New York, NY, USA). Four accelerometers were mounted on the base mass and the fifth was mounted on the top of the suspended mass. A 24-channel 40 kHz bandwidth analyzer (LMS Pimento, Plano, TX, USA) was used to capture the accelerometer’s signals and to generate the sweep sine signals that the shaker converts to sweep sine forces applied to the system.

## 4. Results

In the next subsections, the methodology proposed in Section 2 is applied to the experimental determination of the transmissibility associated to the elastomeric element and the spring element using the experimental setups described in Section 3. These two examples of the application of the methodology are used to validate its correctness and to study its benefits. The background noise initially considered in the simulations is a white noise with a RMS amplitude of 3.3$\times 10^{-10}$ m/s${}^{2}$, which is the average of the ambient vibration at $z_{b}$ measured during usual laboratory operation activities. In both examples, the sweep range considered is 0 Hz to 80 Hz, thus defining the values of $\omega_{e}$ and $\omega_{s}$. From the results of the parametric study, suitable excitation amplitude and sweep rate are chosen based on the estimated error results. Finally, the correctness of the numerical results is verified using experimental measurements.

### 4.1. Application Example I: An Elastomeric Material

Following the methodology presented in Section 2, a parametric study considering the parameters previously stated is performed based on the assumption that the theoretical model presented in Figure 1 properly represents dynamically the experimental setup for the elastomeric material in the range of frequencies between 0 Hz and 80 Hz. Two amplitudes *F* of the excitation forces are assumed in the simulation in order to quantify the effect of the amplitude: 1.5 N and 15 N. It should be noted that, due to the fact that the same background noise is considered for both force amplitude cases, the signal-to-noise ratio (SNR) for the case of 15 N of excitation force amplitude is quite large (about 59 dB) in comparison with the one associated to the 1.5 N amplitude (about 39 dB). The range of sweep rates selected for the study is 0.5 Hz/s to 50 Hz/s. Each simulated measurement consists of five sweep cycles with non-correlated background noise. The simulation is performed in MATLAB, where the time domain solver used is the de45 and the Welch’s method is applied to compute the transmissibility through the algorithm tfestimate with default windowing characteristics (50% of overlap and eight segments).

**Figure 3:**
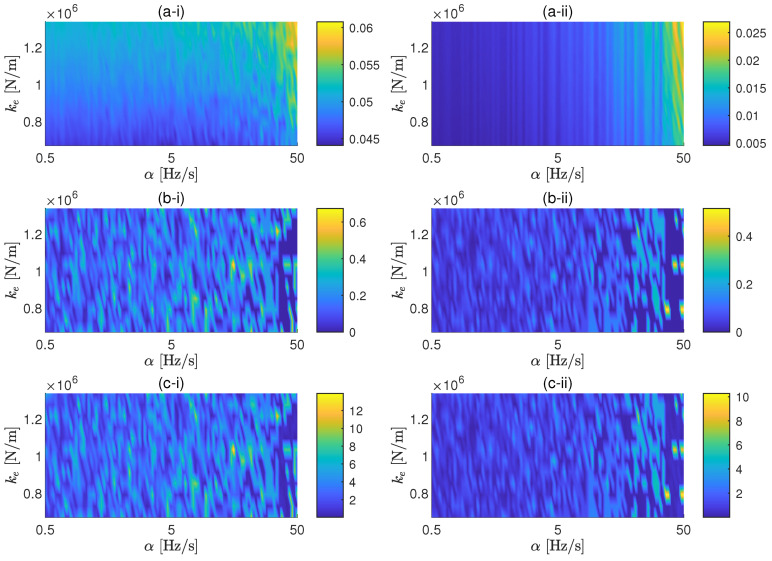
General (**a**), resonance frequency (**b**) and resonance amplitude (**c**) errors as a function of the sweep rates and the dynamic stiffness of the elastomer for excitation force amplitudes of 1.5 N (i) and 15 N (ii). Background noise: 3.3$\times 10^{-10}$ m/s${}^{2}$.

In Figure 3, results of the parametric study are shown. For both force amplitudes, the general error of the FRF is below the 0.06%, although for the force amplitude of 15 N, the errors are smaller and more controlled by the sweep rate. The same tendency appears in the errors of the resonance frequency and peak value, having in the latter quite big errors for large sweep rates, specially for the case of 1.5 N. Because the values for the force amplitude and background noise considered in this case are based on the experimental measurements presented below, it is expected that the errors obtained experimentally resemble the ones predicted in these results (i.e., less that 1% for most sweep rate values).

**Figure 4:**
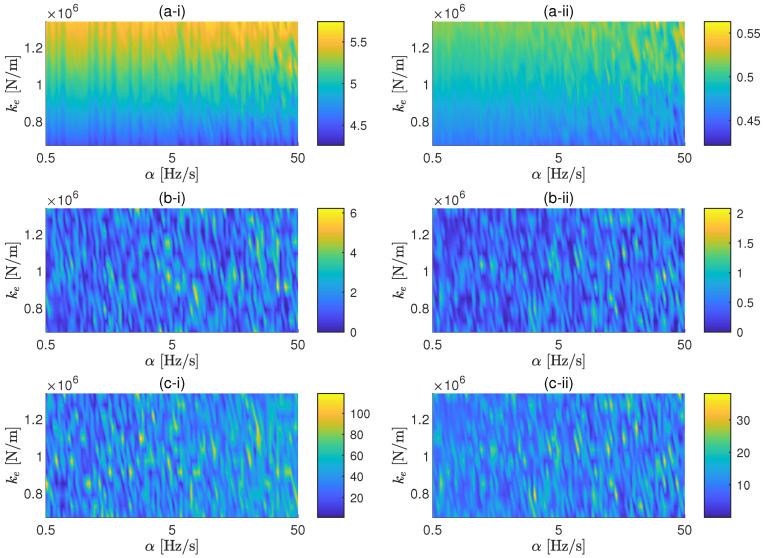
General (**a**), resonance frequency (**b**) and resonance amplitude (**c**) errors as a function of the sweep rates and the dynamic stiffness of the elastomer for excitation force amplitudes of 1.5 N (i) and 15 N (ii). Background noise: 3.3$\times 10^{-8}$ m/s${}^{2}$.

In Figure 4, the results of the parametric study considering a background noise of 3.3$\times 10^{-8}$ m/s${}^{2}$ of average power spectral density, 100 times larger than the background noise measured in the laboratory. As expected, the errors are increasing as the SNR increase (−1 dB for the amplitude of 1.5 N and 19 dB for the amplitude of 15 N). This figure clearly shows that the influence of the sweep rate on the errors of transmissibility in the presence of background noise is almost negligible. Thus, it can be concluded that the lower the signal-to-noise ratio are, the smaller the influence of the sweep rate on the error becomes.

Experimental measurements were performed to determine the sweep rate effect on the experimental estimation of the resilient element transmissibility and compare it with the numerical predictions. As mentioned, four accelerometers were mounted on the base and another one was mounted on the suspended mass. A circular sample of the elastomeric material was placed on the base mass and loaded using steel disks to achieve the required suspended mass. The material sample had a diameter of 200 mm and an initial thickness of 18 mm. Sweep sine signals of constant amplitude with different sweep rates were applied at the base of the test rig. The sweep starting and ending frequencies were 0 and 80 Hz, respectively, and five sweep cycles were considered in each measurement. The total measuring time varied from 10 s (α=40 Hz/s) to 600 s (α=0.67 Hz/s). The amplitude of the sweep excitation was of 1.5 N and was measured using a force transducer mounted on the shaker actuator. In contrast with the numerical case, the exact experimental transmissibility is not known. Due to this, in this application example it is assumed that the transmissibility obtained considering a white noise excitation can be considered as the exact one. Therefore, the response of the system to a random white noise excitation was measured for 600 s, and the resulting transmissibility was used as the reference result in the experimental error calculations presented below. As in the numerical simulation case, the experimental transfer functions were obtained dividing each signal into eight segments with a 50% overlap between segments and a Hamming window was applied to minimise leakage.

**Figure 5:**
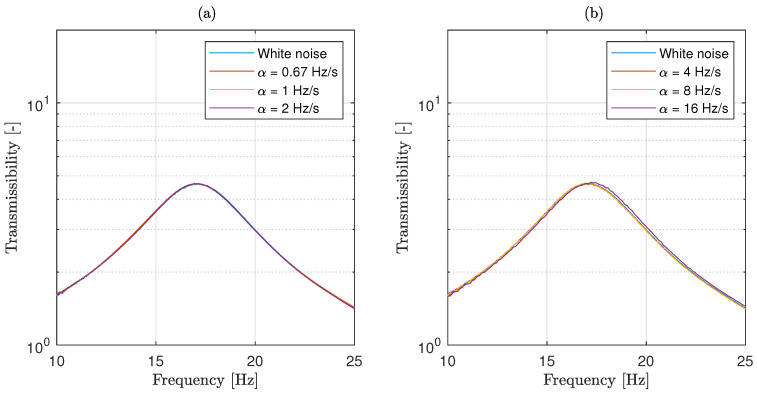
Comparison of the transmissibility obtained using a white noise excitation with those obtained using a sweep sine excitation for the elastomer case, for slower sweep rates (**a**) and faster sweep rates (**b**).

Figure 5 compares the experimental estimation of the system transmissibility for different sweep rates with the transmissibility obtained when a random white noise excitation is considered. As in the numerical simulations case, the hat is used here to refer to the approximate transmissibility. The results show that the peak of the transmissibility curve, which occurs around 17 Hz, is considerably broad, indicating that, as expected, the tested material has a large damping factor. The comparison also shows that, even for the highest sweep rates, there is almost no difference between the transfer functions obtained using a sweep sine excitation and the one obtained considering a random white noise.

**Figure 6:**
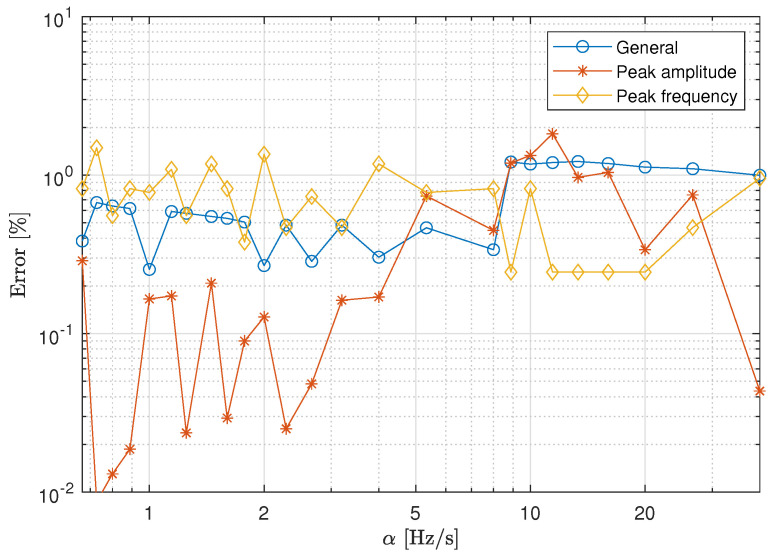
Estimated errors in the experimental transmissibility for the elastomeric material case.

In order to quantify the discrepancies between the transfer functions, the error of the transmissibility estimations using the Equations (5)–(7) is employed. To do so, the transimissibilities obtained using sweep sine excitation are associated to ${\hat{T}}_{sb}$ and the ones obtained due to the white noise excitation to $T_{sb}$. Figure 6 presents a comparison of the three errors as a function of the sweep rate. The results confirm that, even for the highest sweep rate, small differences can be observed between the transfer functions obtained using a sweep sine excitation and those obtained using a white noise excitation. This result can be explained by the large damping factor observed in the transmissibility curves. For a material with high damping, the response decays very fast and the discrepancies arising due to the use of data reduction techniques are negligible [24]. Additionally, due to the smoothness of the transmissibility peak, the maximum amplitude of the curve can be obtained accurately even for very low frequency resolutions (resulting from very short measuring times).

The errors obtained with the experimental measurements are in reasonable agreement with the ones predicted by the numerical simulation that consider a similar excitation amplitude, i.e., the 1.5 N case. The numerical results predicted very small values for the general and peak frequency errors, a result that is also observed in the experimental errors. For the case of the peak amplitude results, the numerical simulations suggest higher discrepancies than the ones observed in the experimental data. This slight difference is attributed to the use of a lower estimation of the real material damping in the simulations. Therefore, it can be concluded that the experimental results validate the use of the numerical model to predict the experimental transmissibility error. The case of a resilient element with much lower damping is presented in the next subsection.

### 4.2. Application Example II: A Coil Spring

**Figure 7:**
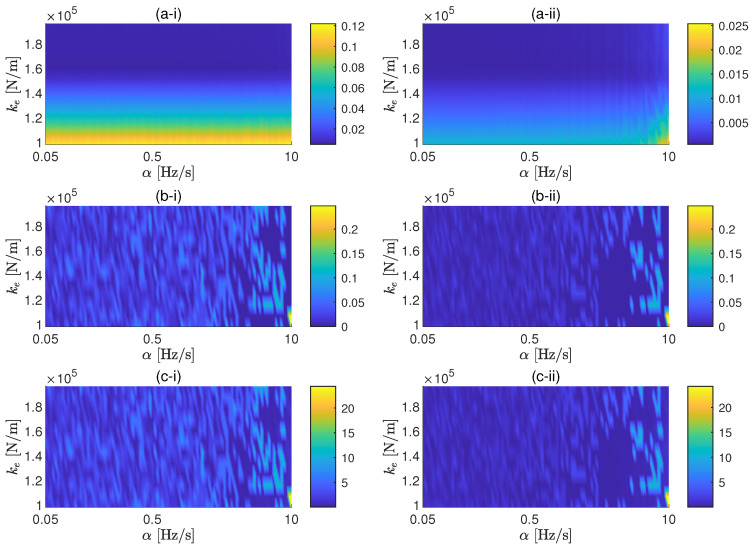
General (**a**), resonance frequency (**b**) and resonance amplitude (**c**) errors as a function of the sweep rates and the dynamic stiffness of the spring for excitation force amplitudes of 2.5 N (i) and 25 N (ii). Background noise: 3.3$\times 10^{-10}$ m/s${}^{2}$.

**Figure 8:**
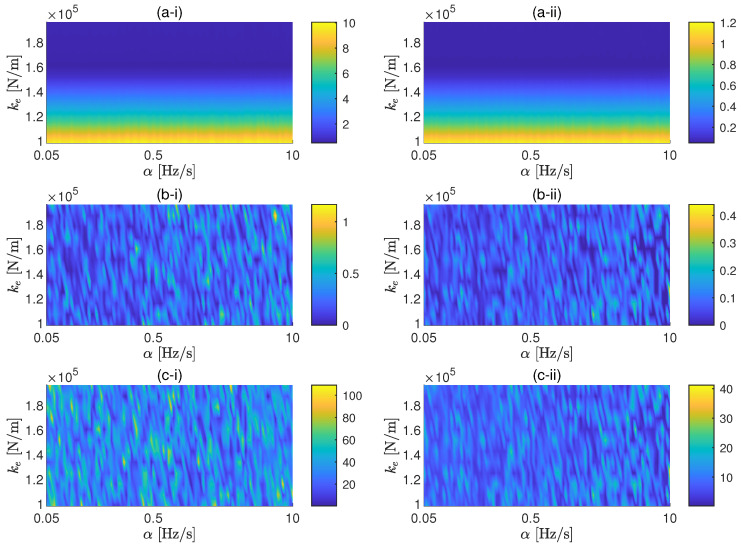
General (**a**), resonance frequency (**b**) and resonance amplitude (**c**) errors as a function of the sweep rates and the dynamic stiffness of the spring for excitation force amplitudes of 2.5 N (i) and 25 N (ii). Background noise: 3.3$\times 10^{-8}$ m/s${}^{2}$.

In this application example, the proposed methodology is applied for a coil spring. The parametric study performed for this case follows the same ideas presented for the case study of an elastomer. The same cases of background noise are also taken in consideration here. In this case, the two values of *F* considered are 2.5 N and 25 N. The associated SNRs for these two values of the force amplitude are approximately 44 dB and 64 dB, respectively, for the low background noise case, and 4 dB and 24 dB, respectively, for the high background noise. The range of sweep rates selected for the study is 0.05 Hz/s to 10 Hz/s. The reason to consider lower sweep rates with respect to the ones considered for the elastomer is because, due to lower stiffness and damping of the spring, the peak of the transmissibility is expected to be located at lower frequencies and to has a sharpener shape. In Figure 7 and Figure 8, the results of the parametric study for the cases of low and high background noise, respectively, are shown. It can be also seen that, due to the sharper shape of the transmissibility peak, the sweep rate affects the FRF in a global sense, as shown by ϵ, only for high SNR. The errors associated to the resonance peak of the transmissibility can be minimised by reducing α for both amplitudes in the case of low background noise, but it is also proven here that the sweep rate cannot efficiently control the error of the FRF in noisy environments.

As in the previous case, experimental measurements were performed to determine the sweep rate effect on the estimation of the spring coil transmissibility and compare the observed discrepancies with the numerical predictions. Steel disks were again used as suspended mass on top of the spring. Sweep sine signals of constant amplitude with different sweep rates were applied at the base of the test rig. In this case, sweep sines with two different excitation ranges where considered: a narrow-band one with starting and ending frequencies of 7 Hz and 14 Hz, respectively, and the previous broad-band sweep from 0 to 80 Hz. This narrower range of frequencies was considered once the transmissibility peak had been identified in order to reduce the length of the acquired time signals. Again, five sweep cycles were considered in each measurement and the total measuring time varied from 50 s (α=8 Hz/s) to 600 s (α=0.67 Hz/s) for the broad-band sweep, and from 100 s (α=0.35 Hz/s) to 600 s (α=0.067 Hz/s) for the narrow-band one. The measured amplitude of the sweep excitation was about 2.5 N. As in the previous case, the response to a random white noise excitation was measured during 600 s and all the transfer functions were processed considering eight segments with a 50% overlap and using a Hamming window.

**Figure 9:**
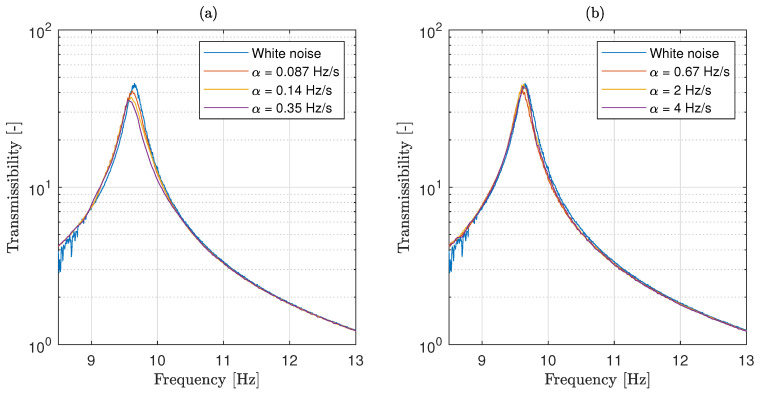
Comparison of the transmissibility obtained using a white noise excitation with those obtained using a sweep sine excitation for the spring coil case, for slower sweep rates (**a**) and faster sweep rates (**b**).

Figure 9 compares the experimental estimation of the mass-spring transmissibility ${\hat{T}}_{sb}$ for different sweep rates with the transmissibility obtained when a random white noise excitation $T_{sb}$ is considered. The results show a sharp peak in the transmissibility curve at 9.7 Hz, suggesting a very low damping value for the spring coil. The results also show some significant discrepancies between the obtained transmissibility curves, specially in the amplitude of curve peaks.

**Figure 10:**
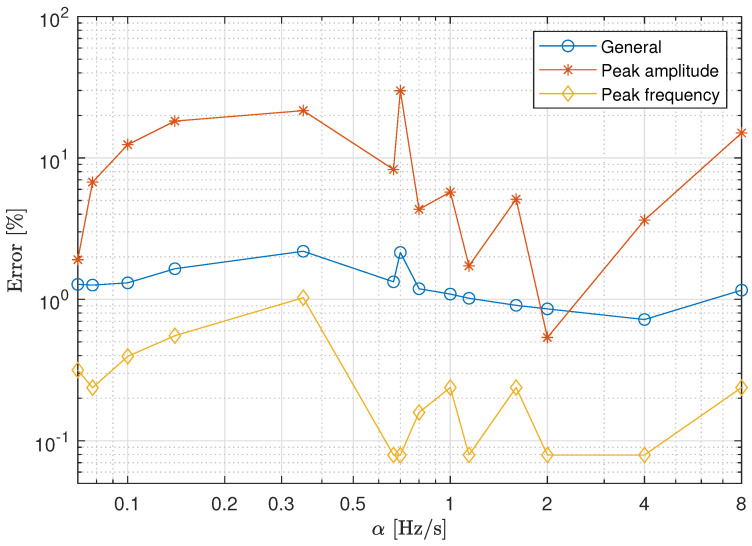
Estimated errors in the experimental transmissibility for the spring coil case.

The discrepancies between the transfer functions ${\hat{T}}_{sb}$ and $T_{sb}$ have been again quantified using Equations (5)–(7). Figure 10 presents a comparison of the three errors as a function of the sweep rate for the spring coil case. The results confirm that the discrepancies between the estimated transmissibility curves are higher than those obtained for the elastomeric material case. This result can be in part attributed to the fact that a high frequency resolution is needed to obtain an accurate estimation of the amplitude of a sharp peak.

The experimental errors agree acceptably well with the numerical predictions for a 2.5 N excitation presented in Figure 7. As in the elastomer case, the numerical model predicted very small values for the general and peak frequency errors, a prediction that is in good agreement with the corresponding experimental estimations. For the case of the peak amplitude error, a good agreement is also observed for high sweep rates. However, unexpectedly large experimental errors have been obtained for low sweep rates. These discrepancies seem to indicate a change in the dynamic behavior of the system when a narrow-band excitation is used. Moreover, the results in Figure 9 show that this change in amplitude is followed by a shift in the peak frequency, a result that suggest a slightly nonlinear response of the tested spring coil.

## 5. Discussion

This paper has proposed a methodology to define the sweep sine excitation parameters to be used when estimating the dynamic properties of an elastomeric material using the indirect method proposed in ISO 10846-3. In the proposed approach, suitable sweep rate and force amplitude values are obtained from parametric studies performed using a simplified numerical model of the test system. The numerical simulations require prior knowledge of the tested sample static stiffness, an average background noise of the laboratory and the test rig parameters.

The proposed methodology has been applied to two resilient elements: an elastomeric material and a coil spring. In both cases, it has been shown that the errors predicted by the numerical simulations agreed well with the error estimations obtained in experimental tests when similar background noise levels where considered. The results have shown that, for low signal-to-noise ratios, the error in the transmissibility is not sensitive to the sweep rate considered.

The results obtained in this work show that the proposed methodology can be specially helpful when the material characterisation is performed in rather noisy laboratories. The methodology could be also extended to characterisation procedures that consider operational conditions, such as in-situ experimental testing. The methodology can be also useful to reduce the setting-up time in cases where several different samples need to be tested, such as in quality control tests in manufacturing process. An additional potential benefit of the proposed approach is that it can be used to define suitable signal processing parameters for each experimental test performed.

## Figures and Tables

**Table 1:** Mechanical parameters of the experimental setups.

Parameters	Value
Base mass ($m_{b}$)	393.5 kg
Stiffness of the $m_{b}$ isolators ($k_{b}$)	29.23 kN/mm
Viscous damping $m_{b}$ isolators ($c_{b}$)	2.265$\times 10^{4}$ Ns/m
Suspended mass ($m_{s}$)	86.23 kg (Elastomer), 39.94 kg (Spring)
Static stiffness of the resilient element ($k_{e}$)	668.4$\times 10^{3}$ N/m (Elastomer), 97.99$\times 10^{3}$ N/m (Spring)
Estimated viscous damping of the resilient element ($\xi_{e}$)	0.05 (Elastomer), 0.01 (Spring)

## Abbreviations

The following abbreviations are used in this manuscript:

FRF	Frequency Response Function
ISO	International Organization for Standardization
SNR	Signal-to-Noise Ratio
